# Right Upper Extremity Idiopathic Primary Lymphedema in a 2-Year-Old Child

**DOI:** 10.7759/cureus.18169

**Published:** 2021-09-21

**Authors:** Victor J Medina, Mohamed S Khalifa

**Affiliations:** 1 Pediatrics, University of Illinois College of Medicine at Peoria, Peoria, USA

**Keywords:** limb swelling, primary lymphedema, hemihypertrophy, pediatric genetics, hematology-oncology

## Abstract

Lymphedema of an extremity at birth can be an alarming finding. Our patient presented with difficulty breathing and productive cough and was found to have primary lymphedema of the right upper extremity since birth. Further testing was mostly unremarkable except for imaging that revealed many splenic lesions.

## Introduction

Meshram et al. state that lymphedema results from the stasis of protein-rich fluid in the interstitial space due to inadequate transport [1]. Lymphedema can either be primary or secondary. While primary lymphedema arises on its own, secondary lymphedema can arise from insults such as infection, surgery, malignancy, and radiation. According to Sleigh and Manna, primary lymphedema is ultra-rare and thought to only affect one in 100,000 individuals [2]. It is known that primary lymphedema is caused by gene alterations that affect the proper development of the lymphatics. Congenital lymphedema is one of the three categories of primary lymphedema. The other two are lymphedema praecox and lymphedema tarda, which occur at puberty or after the age of 35, respectively [2].

## Case presentation

**Figure 1:**
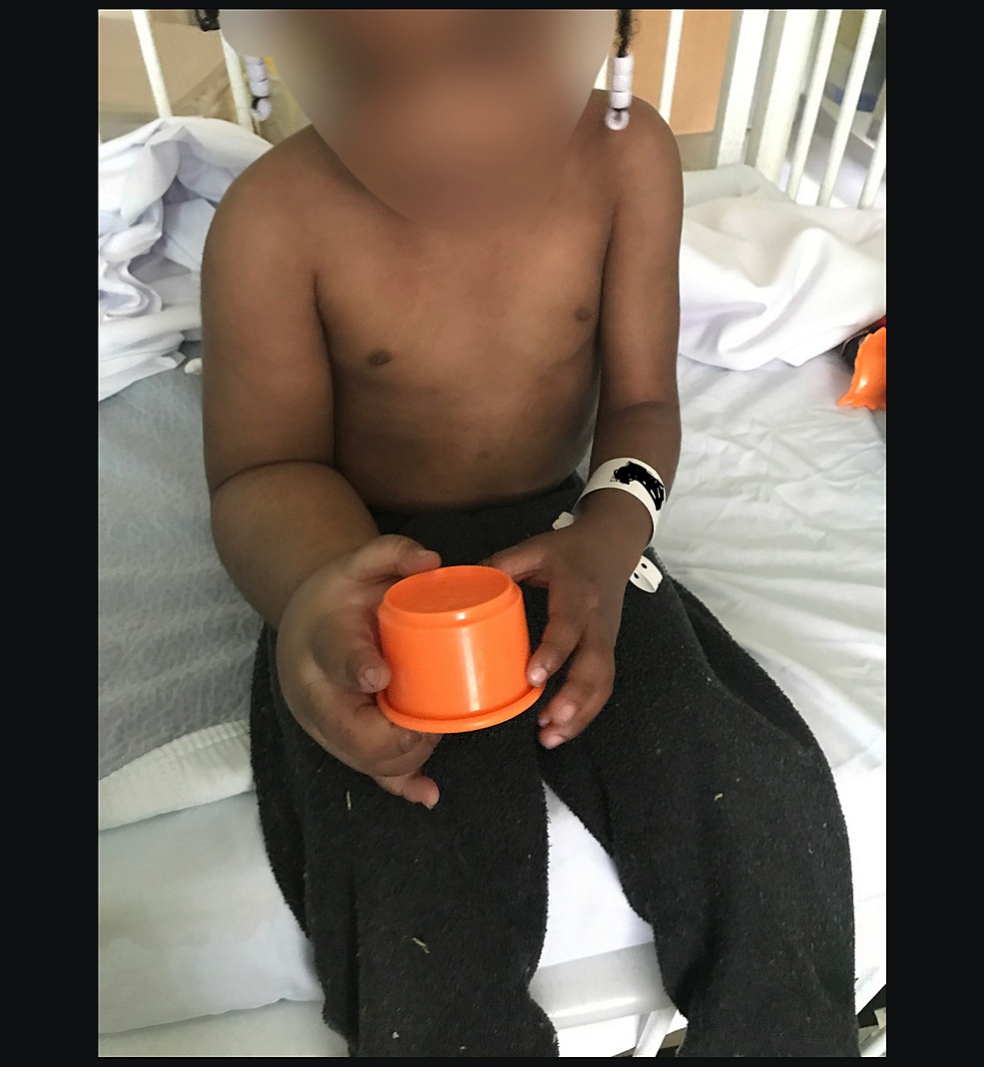
A 2-year-old toddler with unilateral lymphedema of the right upper limb.

Our case is of a 2-year-old toddler with right upper extremity hemihypertrophy and lymphedema that has been present since birth (Figure 1). The patient was admitted to the hospital due to increased difficulty in breathing from an acute asthma exacerbation. The patient’s lymphedema had not been properly worked up due to lost follow-up caused by socioeconomic barriers. During the patient’s hospital stay, our team consulted genetics to assist in evaluating the patient’s lymphedema. The genetics team requested a test for Beckwith-Wiedemann syndrome and Russell-Silver syndrome that showed a decreased likelihood of either disease. Growth differentiation factor 15 was within range. Tumor markers Beta-hCG and alpha-fetoprotein were all within normal limits. A chromosomal microarray showed no clinically relevant copy number variants and no regions with the absence of heterozygosity were observed.

**Figure 2:**
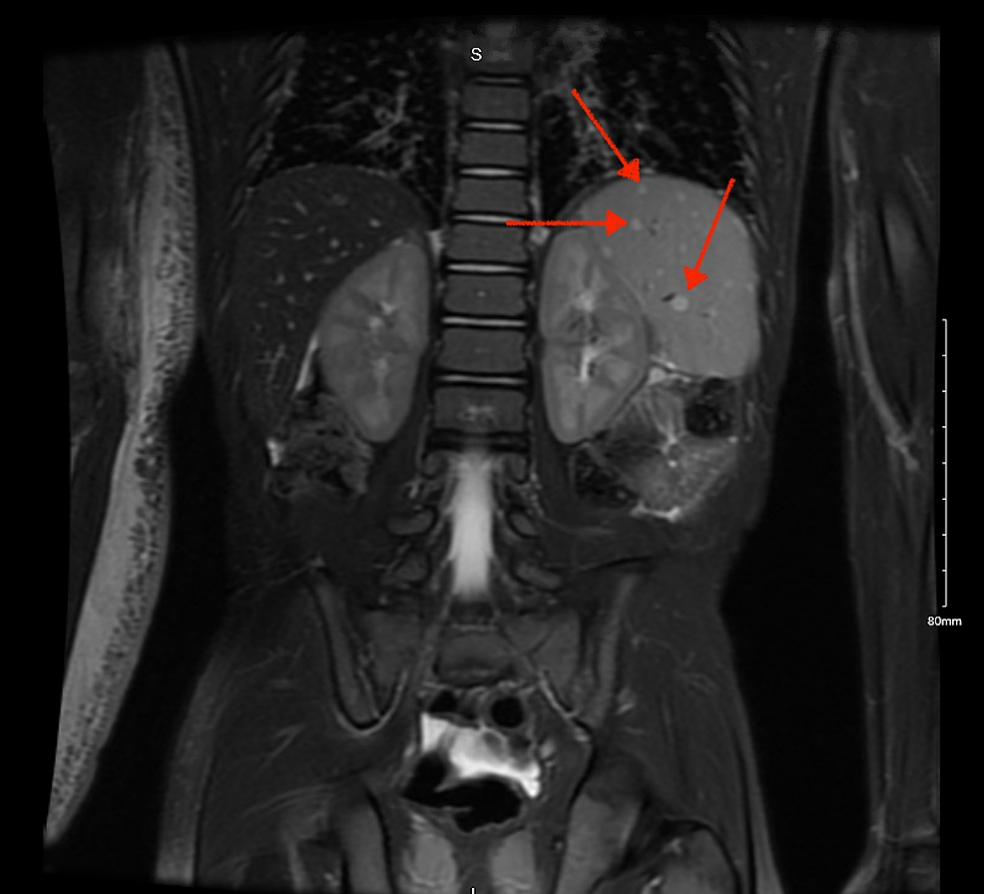
MRI identifying splenic lesions (red arrows) in a 2-year-old with right upper extremity lymphedema

**Figure 3:**
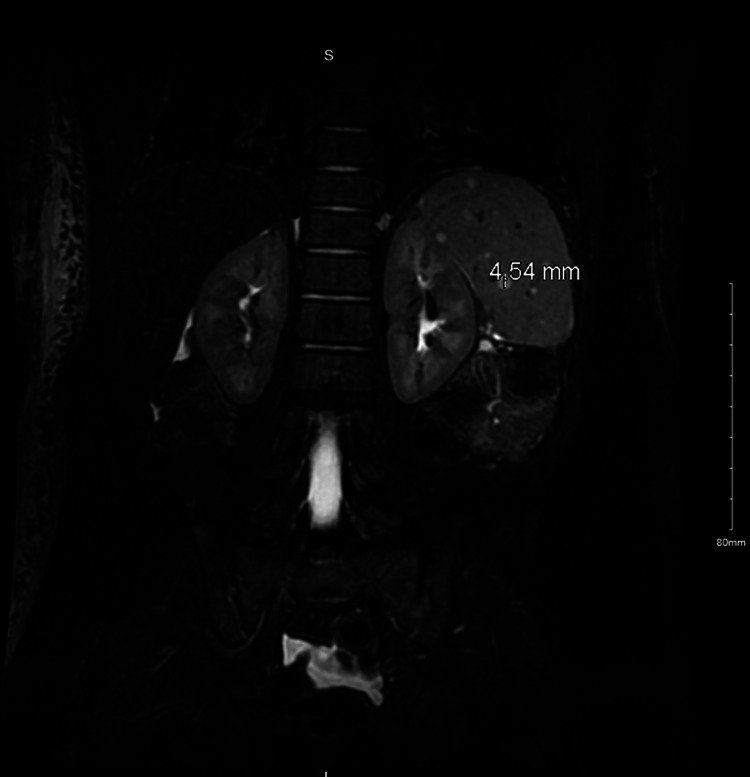
MRI identifying splenic lesions (arrows removed for better visualization) of a 2-year-old with right upper extremity lymphedema

Upon further investigation of the lymphedema, an MRI of the abdomen demonstrated the spleen measured 6.6 cm in length and innumerable T2 hyperintense lesions throughout the entirety of the spleen were found (Figures 2, 3). The largest lesion measured 7 x 5 x 5 mm in size on axial imaging. Although the etiology of these lesions is unclear, they raised suspicion for splenic hemangiomatosis or hamartomas. However, malignancy could not be ruled out. The MRI also demonstrated nonspecific free fluid in the peritoneum and prominently enlarged lymph nodes in the right upper extremity, one measuring 8 x 5 mm in size. Furthermore, a CBC revealed low WBC and elevated hemoglobin and hematocrit. A coagulation profile from the same date showed decreased partial thromboplastin time (PTT).

## Discussion

The purpose of this report was to attempt to determine the cause of congenital lymphedema in a pediatric patient. The patient was seen in the neonatal intensive care unit (NICU) after birth for concerns of his lymphedema, but due to socioeconomic and healthcare barriers, appropriate diagnosis and treatment had been delayed. During his most recent inpatient stay, the pediatric team determined that along with treating the patient's asthma exacerbation, further investigation into the lymphedema was warranted. Our initial suspicion that the congenital lymphedema could possibly be secondary to another disease led us to consult the genetics team. As we awaited the genetic consult there was a concern for Beckwith-Wiedemann syndrome (BWS) and an ultrasound was performed. The ultrasound showed signs of splenic lesions that were concerning. Although BWS is more commonly associated with renal malignancy, a case report by Herm et al. found that a patient with BWS was found to have a splenic hemangioma on routine sonography [3]. Following the results of the ultrasound, a decision to have an MRI of the abdomen was made in order to try and better understand the severity of these lesions. The genetics team then stated that the patient's lymphedema and splenic lesions were unlikely to be from BWS due to negative testing results. Apart from awaiting the results of whole-genome sequencing, a study by Kalawat et al. recommended that patients undergo lymphoscintigraphy, an easy and highly effective imaging technique to evaluate lymphedema of unknown etiology [4]. The complexity of this patient's disease presentation prompted the pediatric team to consult oncology for further investigation of the patient's lymphedema and splenic lesions. The oncology team stated that there is a concern for hemangiomatosis of the spleen, but a splenic biopsy would be required to make the diagnosis. The patient's mother was advised of the high risk of bleeding if a biopsy was done. Oncology also recommended that the child be started on propranolol to try and reduce the lesions or at least prevent them from growing. The patient's mother declined the biopsy and the use of propranolol but agreed to monitor the patient's splenic lesions by abdominal ultrasound every two months until stabilization of the lesions is observed.

## Conclusions

Although primary lymphedema is a rare occurrence, it is important that patients who present with congenital extremity swelling are evaluated promptly, adequately, and efficiently. More importantly, it should be understood that some patients are faced with socioeconomic and healthcare barriers that can delay the appropriate diagnosis and treatment of their disease. There were no signs of malnutrition, undernutrition, or subclinical infection, and based on the results of the extensive workup done on our patient, it is believed that the swelling of his right upper extremity is due to idiopathic primary lymphedema and the splenic lesions were merely an incidental finding.

## References

[1] Meshram GG, Kaur N, Hura KS (2018). Unilateral primary congenital lymphedema of the upper limb in an 11-month-old infant: a clinical and pharmacological perspective. Open Access Maced J Med Sci.

[2] Sleigh BC, Manna B (2021). Lymphedema. https://www.ncbi.nlm.nih.gov/books/NBK537239/..

[3] Herman TE, McAlister PW, Dehner LP, Skinner M (1997). Beckwith-Wiedemann syndrome and splenic hemangioma: report of a case. Pediatr Radiol.

[4] Kalawat TC, Chittoria RK, Reddy PK, Suneetha B, Narayan R, Ravi P (2012). Role of lymphoscintigraphy in diagnosis and management of patients with leg swelling of unclear etiology. Indian J Nucl Med.

